# Supplementary material for: Global CO2 emissions from dry inland waters share common drivers across ecosystems
Source: Nat Commun. 2020 May 1;11:2126. doi: 10.1038/s41467-020-15929-y (PMC7195363; doi:10.1038/s41467-020-15929-y)
Supplement: Supplementary file 1 — Supplementary Information [file 41467_2020_15929_MOESM1_ESM.pdf]

Supplementary Information for

Global CO_2 emissions from dry inland waters share common drivers across ecosystems

by

Keller et al.

Supplementary Tables

Supplementary Table 1: CO_2 fluxes (FCO_2) from dry and inundated inland water systems in the literature. Shown is the mean ± standard deviation.

System, condition and location	FCO_2 (mmol m^{-2} d^{-1})	Reference
**Dry**		
Drawdown area of Nam Theun 2 reservoir, Laos	279 ± 27	Deshmukh et al., (2018) [1]
Drawdown area of CDU reservoir, wet season, Brazil	155	Almeida et al. (2019) [2]
Drawdown area of CDU reservoir, dry season, Brazil	203	Almeida et al. (2019) [2]
Intermittent kettle holes, dry phase, Germany	89 ± 20	Reverey et al., (2018) [3]
Intermittent ponds, annual cycle, Spain	48 ± 36	Catalan et al., (2014) [4]
	149 ± 111	Obrador et al., (2018) [5]
Dry areas of Boadella reservoir, summer, Spain	216 ± 177	Gómez-Gener et al., (2015) [6]
Dry areas of Soyang reservoir, extreme drought, South Korea	515 ± 377	Jin et al., (2016) [7]
Intermittent streams, summer, USA	44 ± 23	Gallo et al., (2014) [8]
Intermittent streams, after rain event, USA	569 ± 530	Gallo et al., (2014) [8]
Intermittent streams, summer, Spain	781 ± 390	Gómez-Gener et al., (2016) [9]
Intermittent stream, summer dry-wet cycles, Australia	72 ± 27	Looman et al. (2017) [10]
Intermittent streams, Spring & Summer, Italy	2.7 – 60.1	Bolpagni et al. (2016) [11]
**Inundated**		
Ponds, global estimate	35 ± 5.21	Holgerson & Raymond, (2016) [12]
Lakes, global estimate	18	Raymond et al., (2013) [13]
Reservoirs, global estimate	28 ± 37	Deemer et al., (2016) [14]
Streams, global estimate	663	Raymond et al., (2013) [13]

Supplementary Table 2: Results from linear mixed effects models of CO_2 emission (FCO_2). Estimates (B) and 95% confidence intervals (CI) are reported. Variables were log_{10}- and z-transformed prior to analysis.

**Fixed parts**	**FCO_2**	
	B	***CI***
(Intercept)	0.04	-0.13 – 0.22
Elevation	-0.22	-0.36 – -0.08
Latitude	0.25	0.05 – 0.44
Conductivity	-0.14	-0.26 – 0.02
Temperature	0.3	0.18 – 0.42
Moisture	0.4	0.26 – 0.51
Organic matter	0.3	0.17 – 0.41
Interaction (Moisture : Organic matter)	0.23	0.14 – 0.32
Interaction (Moisture : Temperature)	0.12	0.02 – 0.22
**Random parts**		
σ	0.47	
σ_{Team}	0.13	

Supplementary Table 3: Global estimate of CO_2 fluxes from dry inland waters. Shown is the mean ± standard deviation.

Type of system	Accumulated dry area during a year (including seasonal and permanent drying) (km^2)	CO_2 emission rate (mmol m^{-2} d^{-1})	Global C emission (Pg C y^{-1})
Streams and rivers	84,461[13]	128 ± 218	0.047 ± 0.081
Lakes, reservoirs	187,542[15]	207 ± 405	0.052 ± 0.1
Ponds	18,390[15]	267 ± 221	0.022 ± 0.018
Total	290,393		0.12 ± 0.13

Supplementary Table 4: Environmental variables used for modeling CO_2 emissions.

Variable	Source	Present in final model
Sediment temperature	Measured *in situ*	
LOI	Measured *in situ*	X
Moisture	Measured *in situ*	X
pH	Measured *in situ*	
Conductivity	Measured *in situ*	X
Texture	Measured *in situ*	
Air temperature	Measured *in situ*	X
Elevation	Determined locally	X
Latitude	Determined locally	X
Type of system	Determined locally	
Annual precipitation	Worldclim[16]	
Annual mean temperature	Worldclim[16]	
Climate zone	Köppen-Geiger[17]	

Supplementary Figures

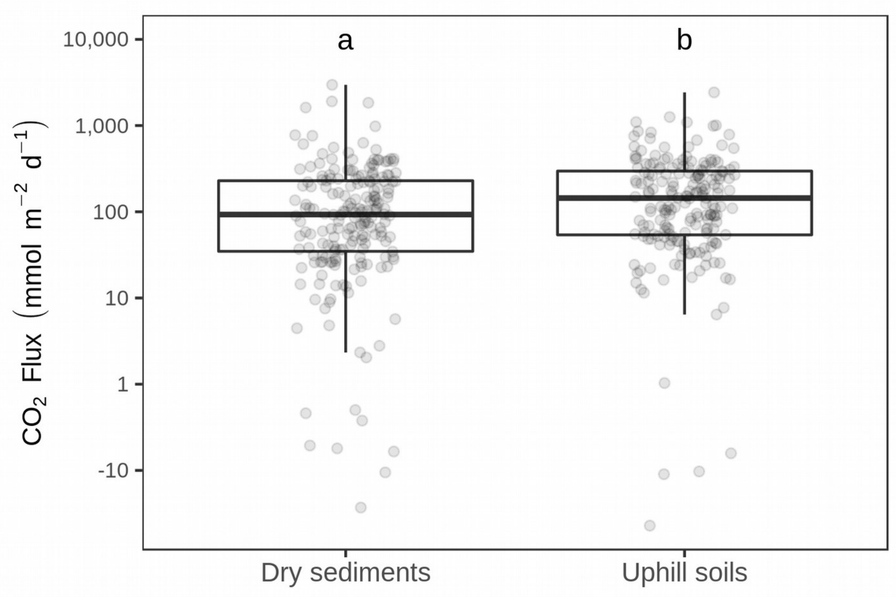

Supplementary Figure 1: CO_2 flux from dry inland waters and uphill soils. Box = 25th and 75th percentiles, whiskers = 1.5 * inter-quartile range. Black line = median. Letters indicate significant differences between systems. (Wilcoxon signed rank test, $P < 0.05$). Note log_{10}-scale in y-axis.

Supplementary References

1. Deshmukh, C. *et al.* Carbon dioxide emissions from the flat bottom and shallow Nam Theun 2 Reservoir: drawdown area as a neglected pathway to the atmosphere. *Biogeosciences* **15**, 1775–1794 (2018).
2. Almeida, R. M. *et al.* Carbon dioxide emission from drawdown areas of a Brazilian reservoir is linked to surrounding land cover. *Aquat. Sci.* **81**, 68 (2019).
3. Reverey, F. *et al.* Dry-wet cycles of kettle hole sediments leave a microbial and biogeochemical legacy. *Sci. Total Environ.* **627**, 985–996 (2018).
4. Catalan, N. *et al.* Carbon dioxide efflux during the flooding phase of temporary ponds. *Limnetica* **33**, 349–359 (2014).
5. Obrador, B. *et al.* Dry habitats sustain high CO_2 emissions from temporary ponds across seasons. *Sci. Rep.* **8**, 3015 (2018).
6. Gómez-Gener, L. *et al.* Hot spots for carbon emissions from Mediterranean fluvial networks during summer drought. *Biogeochemistry* **125**, 409–426 (2015).
7. Jin, H. *et al.* Enhanced greenhouse gas emission from exposed sediments along a hydroelectric reservoir during an extreme drought event. *Environ. Res. Lett.* **11**, 124003 (2016).
8. Gallo, E. L., Lohse, K. A., Ferlin, C. M., Meixner, T. & Brooks, P. D. Physical and biological controls on trace gas fluxes in semi-arid urban ephemeral waterways. *Biogeochemistry* **121**, 189–207 (2014).
9. Gómez-Gener, L. *et al.* When Water Vanishes: Magnitude and Regulation of Carbon Dioxide Emissions from Dry Temporary Streams. *Ecosystems* **19**, 710–723 (2016).
10. Looman, A., Maher, D. T., Pendall, E., Bass, A. & Santos, I. R. The carbon dioxide evasion cycle of an intermittent first-order stream: contrasting water–air and soil–air exchange. *Biogeochemistry* (2016).
11. Bolpagni, R., Folegot, S., Laini, A. & Bartoli, M. Role of ephemeral vegetation of emerging river bottoms in modulating CO_2 exchanges across a temperate large lowland river stretch. *Aquat. Sci.* **79**, 149–158 (2017).
12. Holgerson, M. A. & Raymond, P. A. Large contribution to inland water CO_2 and CH_4 emissions from very small ponds. *Nat. Geosci.* **9**, 222–226 (2016).

13. Raymond, P. A. *et al.* Global carbon dioxide emissions from inland waters. *Nature* **503**, 355–359 (2013).

14. Deemer, B. R. *et al.* Greenhouse Gas Emissions from Reservoir Water Surfaces: A New Global Synthesis. *BioScience* **66**, 949–964 (2016).

15. Marcé, R. *et al.* Emissions from dry inland waters are a blind spot in the global carbon cycle. *Earth-Sci. Rev.* **188**, 240–248 (2019).

16. Fick, S. E. & Hijmans, R. J. WorldClim 2: new 1-km spatial resolution climate surfaces for global land areas. *Int. J. Climatol.* **37**, 4302–4315 (2017).

17. Kottek, M., Grieser, J., Beck, C., Rudolf, B. & Rubel, F. World Map of the Köppen-Geiger climate classification updated. *Meteorol. Z.* **15**, 259–263 (2006).
